# “Metal-bone” scaffold for accelerated peri-implant endosseous healing

**DOI:** 10.3389/fbioe.2023.1334072

**Published:** 2024-01-10

**Authors:** Yue Lu, Xianggang Wang, Hao Chen, Xin Li, He Liu, Jincheng Wang, Zhihui Qian

**Affiliations:** ^1^ Key Laboratory of Bionic Engineering, Ministry of Education, Jilin University, Changchun, China; ^2^ Orthopaedic Medical Center, The Second Hospital of Jilin University, Changchun, China; ^3^ Orthopaedic Research Institute of Jilin Province, Changchun, China

**Keywords:** metal-bone scaffold, Ti6Al4V, 3D-printing, osseointegration, porous scaffolds

## Abstract

Restoring bone defects caused by conditions such as tumors, trauma, or inflammation is a significant clinical challenge. Currently, there is a need for the development of bone tissue engineering scaffolds that meet clinical standards to promote bone regeneration in these defects. In this study, we combined the porous Ti6Al4V scaffold in bone tissue engineering with advanced bone grafting techniques to create a novel “metal-bone” scaffold for enhanced bone regeneration. Utilizing 3D printing technology, we fabricated a porous Ti6Al4V scaffold with an average pore size of 789 ± 22.69 μm. The characterization and biocompatibility of the scaffold were validated through *in vitro* experiments. Subsequently, the scaffold was implanted into the distal femurs of experimental animals, removed after 3 months, and transformed into a “metal-bone” scaffold. When this “metal-bone” scaffold was re-implanted into bone defects in the animals, the results demonstrated that, in comparison to a plain porous Ti6Al4V scaffold, the scaffold containing bone tissue achieved accelerated early-stage bone regeneration. The experimental group exhibited more bone tissue generation in the early stages at the defect site, resulting in superior bone integration. In conclusion, the “metal-bone” scaffold, containing bone tissue, proves to be an effective bone-promoting scaffold with promising clinical applications.

## 1 Introduction

Bone defects often result from various causes such as trauma, tumors, inflammation, and more. Smaller defects can typically heal on their own, but larger bone defects often cannot undergo self-repair (Henkel et al., 2021). In clinical practice, autologous bone transplantation is considered as the gold standard for treating these challenging non-healing bone defects. However, due to the difficulty in sourcing autologous bone, this approach can lead to secondary injuries. Allogeneic bone grafts face issues related to immune rejection (Nayak et al., 2023). To promote bone regeneration at the site of bone defects, bone tissue engineering has emerged as a highly promising technique. Bone tissue engineering generally involves a scaffold, cells, and bioactive substances. Given the specific biological requirements of bone, the scaffold must possess adequate mechanical strength, excellent biocompatibility, and osteoconductivity (Wang et al., 2020). Hence, Ti6Al4V scaffolds have found wide application in bone tissue engineering. Nevertheless, since pure Ti6Al4V scaffolds can merely fill defects and have limited potential to enhance bone regeneration, it is essential to incorporate osteoinductive components into the scaffold (Li et al., 2023). Considering that the gold standard for bone defect treatment in clinical practice is bone transplantation, the development of a novel “metal-bone” scaffold that combines bone tissue with a metal scaffold holds the potential to significantly enhance bone regeneration outcomes.

Titanium alloy, Ti6Al4V, is a commonly used biomaterial in orthopedic surgery. Titanium alloys are favored due to their excellent biocompatibility, osseointegration properties, higher strength, and corrosion resistance compared to pure titanium (Gu et al., 2022; Liu et al., 2022). However, as a type of alloy, Ti6Al4V possesses a significantly higher strength than human bone tissue. Consequently, when Ti6Al4V implants are placed inside the body, stress shielding phenomena occur, leading to stress concentration and hindering optimal bone healing (Naghavi et al., 2023b). To address this issue, the current consensus suggests the use of porous Ti6Al4V scaffolds, effectively reducing the relative strength of the scaffold and mitigating stress shielding problems (Abbasi et al., 2020).

The preparation of porous Ti6Al4V scaffolds commonly involves the use of 3D printing technology, a method prevalent in current research (Subasi et al., 2023). 3D printing, also known as additive manufacturing, is a technology that fabricates three-dimensional objects layer by layer based on three-dimensional models. Utilizing 3D printing technology, it is possible to precisely and conveniently manufacture Ti6Al4V scaffolds with specific porous structures (Altunbek et al., 2023).

In previous research, extensive studies have been conducted on the optimal pore size of porous Ti6Al4V scaffolds for promoting osteogenesis (Zhang et al., 2022). Research has shown that pores that are too small (<400 μm) can lead to excessive scaffold strength, severe stress shielding, and hinder the ingrowth of new bone tissue (Chen et al., 2020). Conversely, pores that are too large (>1,000 μm) can result in insufficient scaffold strength, providing ineffective mechanical support, and impeding bone tissue ingrowth, ultimately causing loosening of the implant at the bone-tissue interface (Wang et al., 2021a). Some research findings have suggested that a pore size around 600 μm is considered optimal (Wang et al., 2017). However, for titanium alloy, strength is a crucial consideration as well. Smaller pores might lead to excessive strength and serious stress-shielding effects, while larger pores can mitigate stress-shielding effects (Zeng et al., 2022; Naghavi et al., 2023a). Taking into account both scaffold strength and pore size, in this study, 3D-Printed Ti6Al4V porous scaffolds were designed with a pore size of 800 μm, consistent with the previously established optimal pore size from experimental research (Wang et al., 2021b). The porosity was set at 70%, aligning with the porosity found in physiological bone trabeculae, ensuring the best osteogenic outcomes (Cui et al., 2021).

**SCHEME 1:**
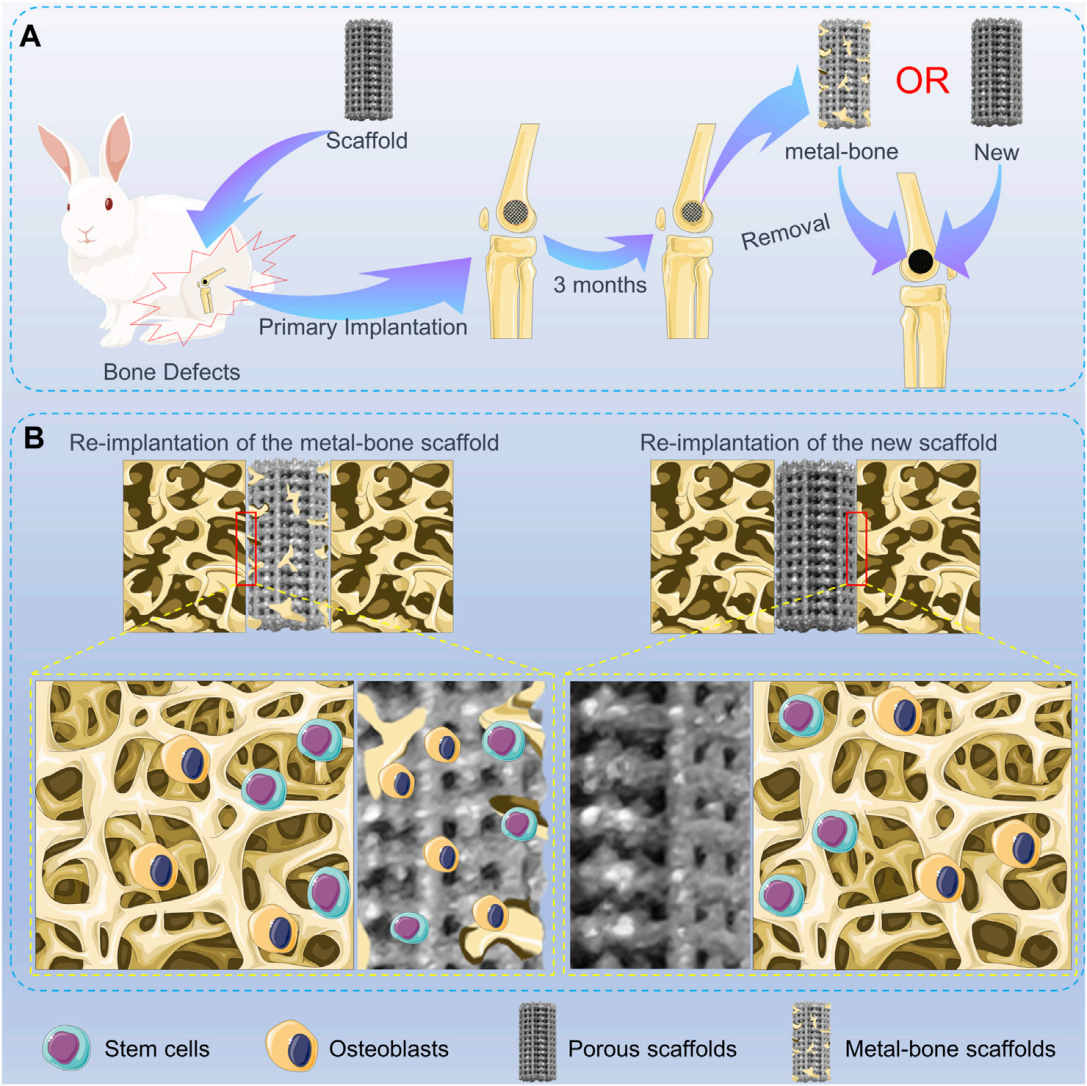
Schematic illustration of the metal-bone and new scaffolds in bone defects. **(A)** Animal experiments of metal-bone implants and new implants. **(B)** The interface between two types of implants and surrounding bone tissue.

In this study, a novel “metal-bone” scaffold was developed for the treatment of bone defects. Initially, a porous Ti6Al4V scaffold was 3D printed. Subsequently, the scaffold was implanted into the bone defect site in experimental animals. After a sufficient amount of bone tissue had grown inside the scaffold, the scaffold was removed from the bone defect site, resulting in the formation of the “metal-bone” scaffold. This “metal-bone” scaffold was then implanted into the bone defect and compared with a control group using a porous Ti6Al4V scaffold without bone tissue, to evaluate the ultimate effects on bone regeneration and integration, as illustrated in Scheme 1.

## 2 Materials and methods

### 2.1 Materials

The Ti6Al4V powder was obtained from AK Medical Co., Ltd. (Beijing, China). The low Glucose Dulbecco’s Modified Eagle’s Medium (DMEM), streptomycin–penicillin dual Antibiotics, and fetal bovine serum (FBS) were purchased from Gibco (Grand Island, NY, United States). Pareformaldehyde and Phosphate buffer (PBS) were obtained from Solarbio (Beijing, China). The Live-Dead staining kit was obtained from Bioss (Beijing, China). The Hematoxylin and Eosin (H&E) stain, Masson’s trichrome stain, and Van Gieson (VG) stain were purchased from Thermo Fisher Scientific (Shanghai, China). Rhodamine phalloidin and 4′,6-diamidino-2-phenylindole (DAPI) were obtained from Thermo Fisher Scientific (Shanghai, China). The double-distilled water used in this study was obtained from a Milli-QA10 filtration system (Milipore, Billerican, MA, United States). BMP-2 and OCN antibodies used in immunofluorescence were purchased from Abcam (Cambridge, United Kingdom).

### 2.2 Preparation and characterization of the 3D-printed porous Ti6Al4V scaffolds

The preparation method for the 3D-Printed porous Ti6Al4V scaffolds utilized in this study is consistent with the description provided in previously published articles (Bai et al., 2020). In brief, we initiated the process by creating a three-dimensional cylindrical model with a specified diameter of 5 mm and a height of 10 mm. Key parameters were set to include a pore size of 800 μm, a porosity of 70%, and a strut diameter of 300 μm. The material employed for printing the scaffolds was biomedical-grade Ti6Al4V powder, and the 3D printing equipment used was an Electron Beam Melting (EBM) printer (Q10, Arcam, Sweden). The scaffolds, characterized by a uniform pore structure, were printed layer by layer. Following the printing process, all scaffolds underwent ultrasonic cleaning to remove any unadhered Ti6Al4V powder from their surfaces. Subsequently, they were subjected to repeated cleaning with acetone, alcohol, and deionized water. Prior to cell experiments and *in vivo* implantation, the scaffolds were sterilized through high-pressure autoclaving. To validate the fidelity of the final printed scaffolds to the design specifications, post-printing visual inspections of scaffold morphology were conducted. Optical microscopy (Olympus Ⅸ, Japan) was employed to magnify observations, and scanning electron microscopy (SEM, JEOL, Tokyo, Japan) was utilized to scrutinize surface topography. Energy-dispersive X-ray spectroscopy (EDS, Aztec software, Oxford Instruments, Abingdon, United Kingdom) analysis was then performed to qualitatively assess the elemental composition of the material, ensuring the absence of impurities or contamination during the fabrication process.

To assess the surface hydrophilicity of the printed scaffolds, water contact angle measurements were conducted in this study using a water contact angle goniometer (DM-500, Kyowa Interface Science Co., Ltd.) Initially, the 3D-Printed Ti6Al4V scaffolds to be tested were prepared, ensuring that their surfaces were dry, clean, and free from impurities. A microliter syringe was employed to dispense a droplet of deionized water onto the material surface, and the shape of the water droplet on the material surface was recorded using the goniometer’s integrated camera. Subsequently, the water contact angle was measured from the images using ImageJ 1.53c software (National Institutes of Health, Bethesda, MD, United States). Three samples were tested, with each sample subjected to three repeated measurements to obtain a reliable average contact angle value.

### 2.3 Extraction and cultivation of bone marrow mesenchymal stem cells

The method for extracting bone marrow mesenchymal stem cells (BMSCs) was as follows: New Zealand White rabbits aged 1 week were selected. The long bones of the rabbit’s limbs were extracted, and bone marrow was flushed from the long bones using a sterile 1 mL syringe and sterile PBS. The flushed cells were then cultured in low-glucose DMEM medium containing 10% fetal bovine serum and 1% penicillin-streptomycin. The cell culture dishes were placed in a constant temperature incubator for cell cultivation. On the third day of cultivation, the cells in the culture dish underwent partial medium replacement, and subsequently, the medium was changed every 3 days. When the cells reached 80%–90% confluence, they were passaged after digestion with trypsin. In this study, the BMSCs used were all from the third generation or higher of mesenchymal stem cells.

### 2.4 Live/dead staining

In a 12-well plate, 40,000 BMSCs were seeded in each well. In the control group, only an equivalent number of BMSCs were seeded into the respective wells. In the experimental group, in addition to seeding an equivalent number of cells, sterile and disinfected Ti6Al4V porous scaffolds were placed in the corresponding wells of the plate. The plate was then placed in a constant temperature incubator for 24 h of cultivation, after which a live-dead staining assay was conducted.

Initially, the plate was centrifuged at 3,000 rpm for 2 min, and the culture medium was aspirated. Live staining solution was added, and the plate was returned to the constant temperature incubator for 30 min of staining. After live staining, the wells were washed three times with PBS. Subsequently, dead staining solution was added, and the plate was stained for 5 min. After staining was completed, the wells were again washed three times with PBS. The live-dead staining results were observed under a fluorescence microscope (Olympus IX71, Tokyo, Japan). All staining and observation procedures were conducted under subdued light conditions.

### 2.5 Immunofluorescence staining of the cell cytoskeleton

To observe the influence of Ti6Al4V porous scaffolds on the cellular morphology of BMSCs, in this study, staining and observation of the BMSCs’ cytoskeleton were conducted using Rhodamine-labeled phalloidin staining solution. Initially, 10,000 BMSCs were seeded in each well of a 12-well plate. In the experimental group, Ti6Al4V porous scaffolds were placed in the corresponding wells, while the control group contained cells only. After 3 days, the staining and observation were performed as follows.

First, the culture medium in the wells was aspirated, and fixation was carried out using a 4% paraformaldehyde solution for 10 min. Subsequently, the cells were washed three times with PBS. Then, they were subjected to light-protected staining with Rhodamine-phalloidin staining solution for 30 min. After 30 min, the cells were washed three times with PBS. DAPI staining solution was applied for light-protected staining for 5 min. Following staining, the cells were washed three times with PBS. After the final washing, the staining results were observed using a fluorescence microscope. All procedures involving staining and observation were conducted under subdued light conditions.

### 2.6 Initial implantation of scaffolds

All animal experimental protocols were approved by the Animal Care and Use Ethics Committee of Jilin University (2022142). Twenty-four adult male New Zealand rabbits were selected for this study. The animals were anesthetized via intravenous injection of 0.5% pentobarbital sodium into the marginal ear vein. The surgical site was selected as the right distal femur for implantation. Prior to surgery, the fur around the knee joint area was shaved using clippers. During the surgical procedure, the rabbits were secured on the operating table. After standard sterilization procedures, a 1–2 cm incision was made on the outer edge of the rabbit’s distal femur using a scalpel. Hemostatic forceps were used for blunt dissection of the subcutaneous muscles, ligaments, and blood vessels around the distal femur, exposing the lateral condyle. A specialized orthopedic core drill with an inner diameter of 4 mm and an outer diameter of 5 mm was then used to create a borehole with a depth of 10 mm, resulting in a bone defect matching the size of the printed scaffold precisely.

The bone debris at the defect site was carefully removed and the defect was rinsed with physiological saline. Subsequently, the scaffold was implanted into the distal femur. The incision was closed layer by layer, with absorbable sutures used for suturing muscles, tendons, ligaments, etc., in the inner layer, and non-absorbable sutures for suturing the skin in the outer layer. Postoperatively, penicillin was administered for 3 days, and the animals’ condition was observed for 1 week.

### 2.7 Secondary implantation surgery

After 3 months from the initial implantation surgery, the same anesthesia, shaving, and sterilization procedures were performed on the rabbits. The 24 rabbits were randomly divided into two groups: the control group with the new scaffolds (Con) implanting consisting of 12 rabbits, the experimental group with the “metal-bone” scaffolds (MB) implanting group consisting of 12 rabbits. During the surgery, the Ti6Al4V scaffold from the right hind limb’s distal femur was first removed. For the MB group, the removed scaffold, which contained bone tissue, was re-implanted into the same rabbit’s opposite hind limb at an equivalent anatomical position in the distal femur. For the control group, after removing the scaffold from the right side, an identical new Ti6Al4V porous scaffold was implanted into the defect site in the other hind limb. Similar to the initial surgery, the incision was closed layer by layer. Postoperatively, penicillin was administered for 3 days to prevent infection, and the rabbits were closely observed for 1 week.

The scaffolds containing bone tissue, taken from the control group, were subjected to Micro-computed tomography (Micro-CT) scans (SkyScan 1076 scanner, Bruker Micro-CT NV, Kontich, Belgium), SEM observation, and mechanical testing. The mechanical testing was conducted using a universal testing machine (H25KS, Hounsfield, United Kingdom), with the scaffolds placed on the sample platform of the testing machine. Compression tests were performed at a rate of 1.0 mm/min until the scaffolds reached their maximum force and maximum deformation. The maximum compressive strength and maximum force that the Ti6Al4V scaffolds with and without bone tissue could withstand were recorded. The mechanical performance differences between the “metal-bone” scaffolds and the new scaffolds were compared.

### 2.8 Micro-CT analysis

At 6 and 12 weeks post the second implantation surgery, euthanasia of the rabbits was carried out using carbon dioxide asphyxiation. The left femoral scaffold implantation sites were then harvested for evaluation. Micro-CT scans were performed to assess bone regeneration at the implantation sites. A cylindrical region of interest with a diameter of 5 mm and a height of 10 mm was selected for three-dimensional reconstruction and subsequent analysis of bone tissue parameters. Specific analyses included volume/tissue volume ratio (BV/TV, %), trabecular thickness (Tb.Th, mm), trabecular separation (Tb.Sp, mm), and trabecular number (Tb.N, 1/mm).

### 2.9 Hard sectioning and staining

Following the completion of Micro-CT scanning, the specimens were fixed in 4% formalin solution. Subsequently, the scaffold implantation sites were subjected to hard sectioning and staining using Masson’s trichrome staining and Van Gieson (VG) staining. Stained sections were then observed and photographed under an optical microscope.

### 2.10 H&E, Masson’s trichrome, and immunohistochemical staining

After fixation, the specimens were subjected to decalcification using 10% EDTA. Following decalcification, sections were prepared using a microtome and subjected to H&E, Masson’s trichrome, and immunohistochemical staining. The specific procedures were consistent with those described in previously published articles for immunohistochemical staining of osteogenic-related genes in the scaffold implantation sites. In brief, bone tissue sections were incubated with BMP-2 and OCN antibodies overnight at 4°C. Subsequently, the samples were washed three times with PBS. The scaffold implantation sites in the sections were then observed and photographed under an optical microscope. The expression intensity of the relevant proteins around the scaffold was quantitatively analyzed using image analysis software ImageJ.

### 2.11 Push-out tests

A standard mechanical push-out test was conducted to assess the interfacial strength between the scaffold and the surrounding bone tissue in each group. Mechanical push-out tests were performed using a universal testing machine. Initially, the samples were placed on the test platform for the mechanical push-out test, with a displacement rate of 1.0 mm/min. The force applied during the scaffold push-out process was recorded using software, with the endpoint being when the scaffold completely disengaged from the bone. By recording the maximum push-out force during the experiment, the interfacial strength between the scaffold and the surrounding tissue was validated.

### 2.12 Statistical analysis

All data are presented as the mean ± standard deviation of at least three independent experiments. Statistical analysis was performed using *t*-test followed by *post hoc* multiple comparison tests using SPSS 19.0 software (SPSS Inc., Chicago, Illinois, United States) to determine the minimum significant differences. Statistical significance was considered at *p* < 0.05.

## 3 Results and discussion

### 3.1 Characterizations of the 3D-printed porous scaffolds

**FIGURE 1:**
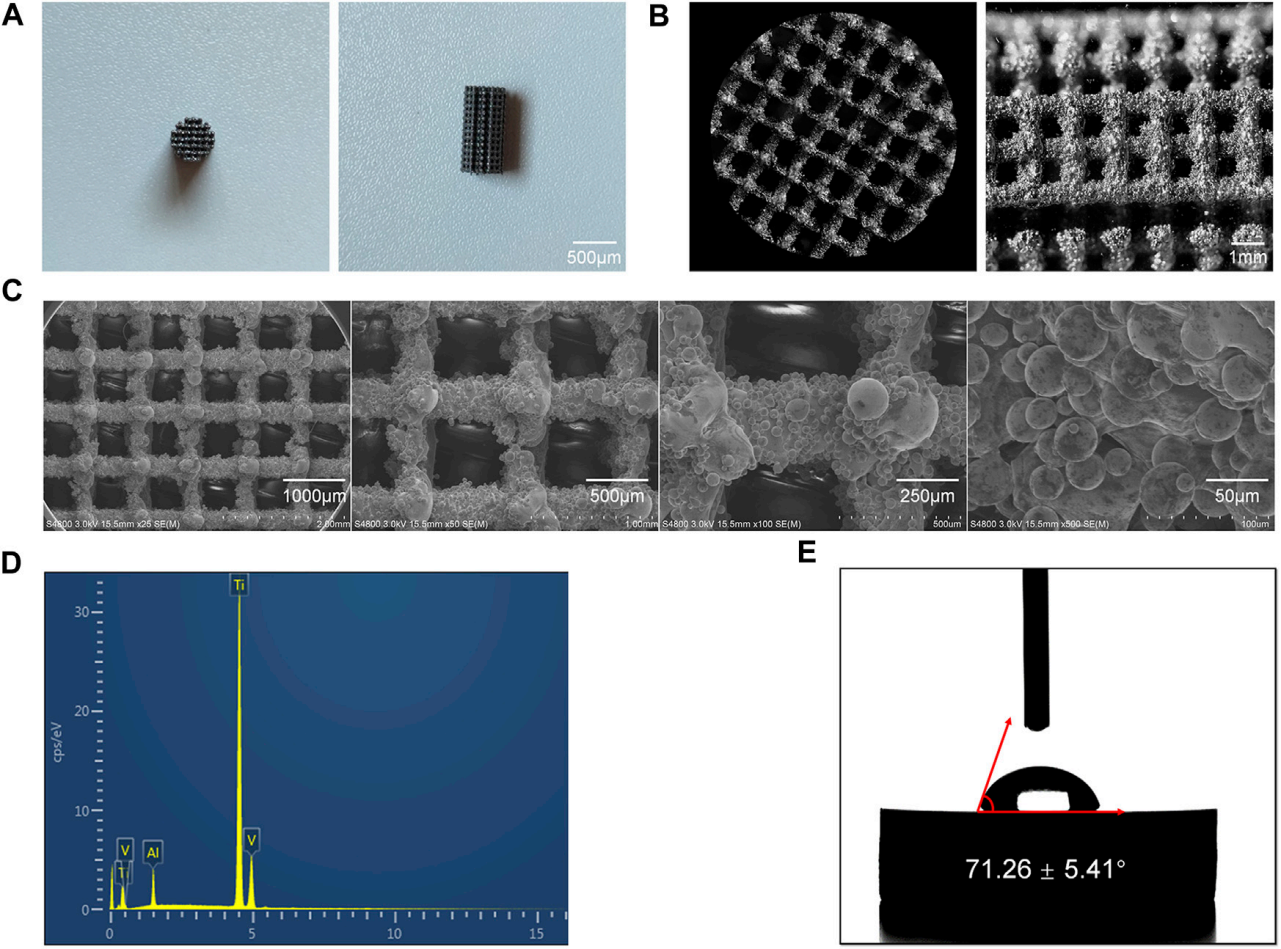
Characterization of the 3D-Printed Ti6Al4V porous scaffolds. **(A)** General appearance of the scaffold. **(B)** Microscopic images of the scaffold. **(C)** SEM images of the scaffolds at various magnifications. **(D)** Elemental composition of the Ti6Al4V scaffolds. **(E)** Water contact angle measurements of the Ti6Al4V scaffolds.

The final 3D-Printed Ti6Al4V porous scaffold exhibited the characteristics shown in Figures 1A, B. It is evident that the scaffold, matching the model parameters, took the form of a porous cylinder with a diameter of 5 mm and a height of 10 mm. This scaffold’s dimensions were entirely consistent with the bone defect size prepared for the subsequent animal experiments. Furthermore, as shown in Figure 1C from the scanning electron microscopy results, during the printing process of the scaffold, it was primarily formed through the fusion of powder particles. The scaffold’s surface was filled with Ti6Al4V particles of varying sizes, resulting in a rugged surface structure.

On one hand, this rough surface structure enhances the adhesion of cells to the scaffold, particularly the migration and adhesion of mesenchymal stem cells, thereby promoting the bone formation process (Montero et al., 2020). On the other hand, the uneven surface structure provides a certain degree of fixation, making it more favorable for the scaffold to be firmly secured in the defect site, preventing implant displacement (Calore et al., 2023). From the scanning electron microscopy results, it can be observed that the pore size of the final printed scaffold was 789 ± 22.69 μm, which closely matched the parameters designed before printing.

Through EDS analysis, as shown in Figure 1D, the results indicated that the main component of this scaffold was Ti6Al4V. There were no impurity elements detected during the scaffold’s preparation, meeting the clinical implantation requirements.

Furthermore, the hydrophilicity of the scaffold’s surface was assessed using a water contact angle measurement device, with results displayed in Figure 1E. It can be observed that the water contact angle on the surface of the 3D-Printed Ti6Al4V porous scaffold was 71.26° ± 5.41°. In previous studies, when the water contact angle is less than 90°, it is considered that the material surface has a certain degree of hydrophilicity. A smaller water contact angle indicates better surface hydrophilicity. Good hydrophilicity suggests that the scaffold possesses excellent biocompatibility and promotes cell adhesion (Sokoot et al., 2023).

In this study, the 3D-Printed porous Ti6Al4V scaffold exhibited a water contact angle of less than 90°, indicating good surface hydrophilicity. This characteristic is beneficial for the adhesion of mesenchymal stem cells after implantation *in vivo*.

### 3.2 Cell viability and morphology

**FIGURE 2:**
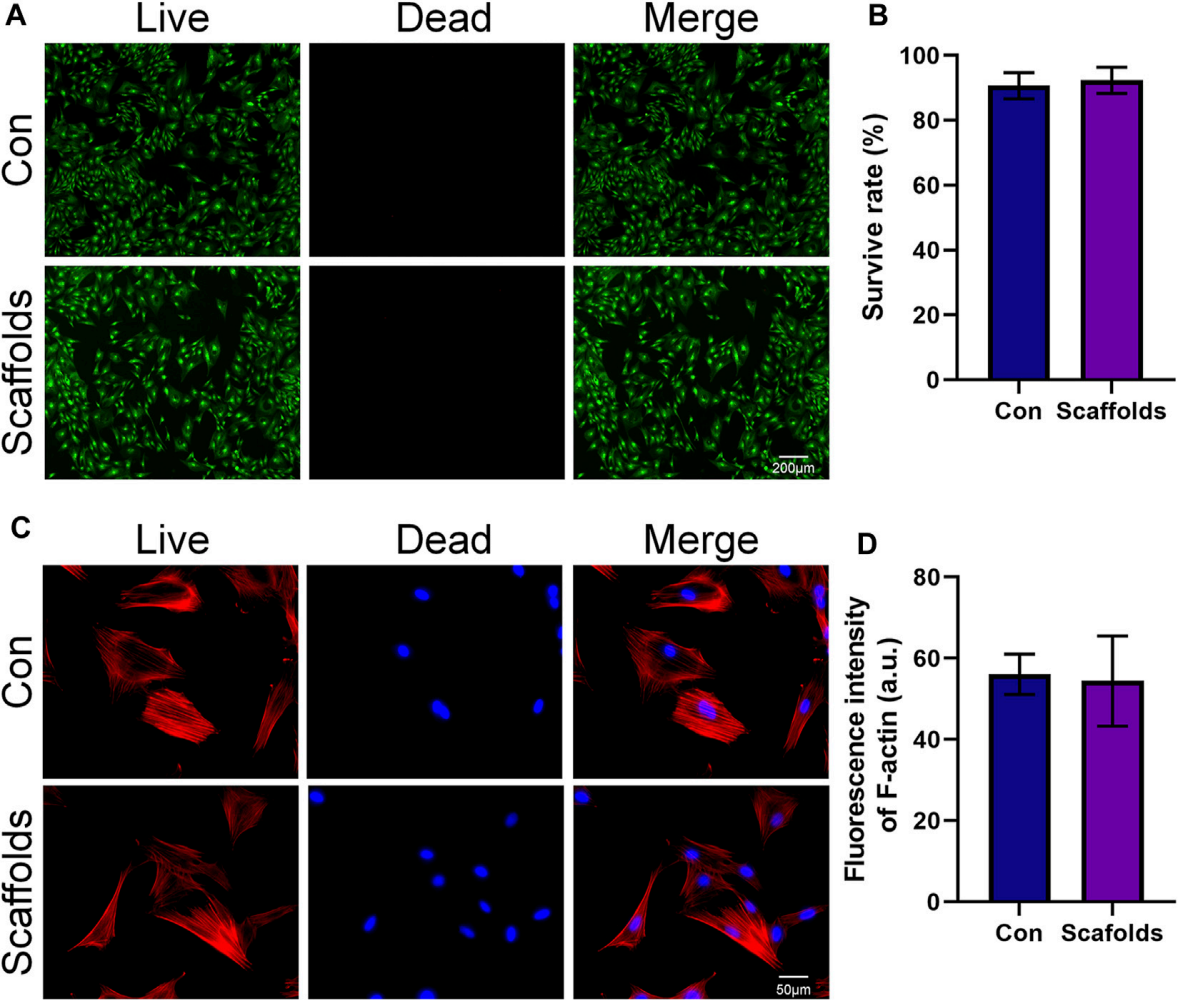
Biocompatibility of the porous scaffolds. **(A)** Calcein AM/PI staining of live cells (green) and dead cells (red). **(B)** Quantitative analysis of cell survival rate (*n* = 3). **(C)** Fluorescent images of the cellular morphology. **(D)** Quantitative analysis of the fluorescence intensity in different groups (*n* = 3). The data are expressed as the mean ± SD. *indicates significant differences between groups. *p < 0.05, **p < 0.01, and ***p < 0.001.

To validate the biocompatibility of the scaffold, it was co-cultured with the most commonly used orthopedic cells, BMSCs. The influence of the scaffold on BMSC growth was assessed by monitoring cell viability and morphology. The results of live-dead staining are shown in Figure 2A. It can be observed that, compared to the control group where BMSCs were growing normally, the addition of the scaffold did not significantly affect the viability of BMSCs, as indicated by the statistical results in Figure 2B. Quantitative analysis revealed that adding the scaffold did not impact the cell survival rate, suggesting that the scaffold material itself does not affect the normal survival of BMSCs.

Microfilaments are important components of the cell cytoskeleton (Jockusch et al., 2004; Muranova et al., 2022). In this study, immunofluorescence staining was used to observe cell morphology by staining the microfilaments in BMSCs. The results are displayed in Figures 2C, D. The F-actin filaments were stained by rhodamine-phalloidin in red, and nuclei were stained by DAPI in blue. It can be observed that in the control group, BMSCs exhibited good spreading, and the microfilament morphology within the cells was clear. In the experimental group, BMSCs adhered well to the scaffold surface. While their cell size appeared slightly smaller, they exhibited longer extensions compared to the control group. The cells on the scaffold showed regular morphology, with a distinct spindle-shaped structure and longer pseudopods. Similar to the control group, the microfilament morphology within the cells was clear. This result suggests that the 3D-Printed porous Ti6Al4V scaffold used in this study allows BMSCs to effectively adhere to its surface. The adhered BMSCs exhibit typical cell morphology, and the cell cytoskeleton is clearly distinguishable.

Taken together, the results of live-dead staining and cell cytoskeleton staining demonstrate that the 3D-Printed Ti6Al4V scaffold used in this study exhibits excellent biocompatibility. It does not adversely affect the survival of BMSCs, and the scaffold surface promotes cell adhesion and spreading without affecting cell morphology.

### 3.3 Osseointegration of the porous scaffold with surrounding bone

**FIGURE 3:**
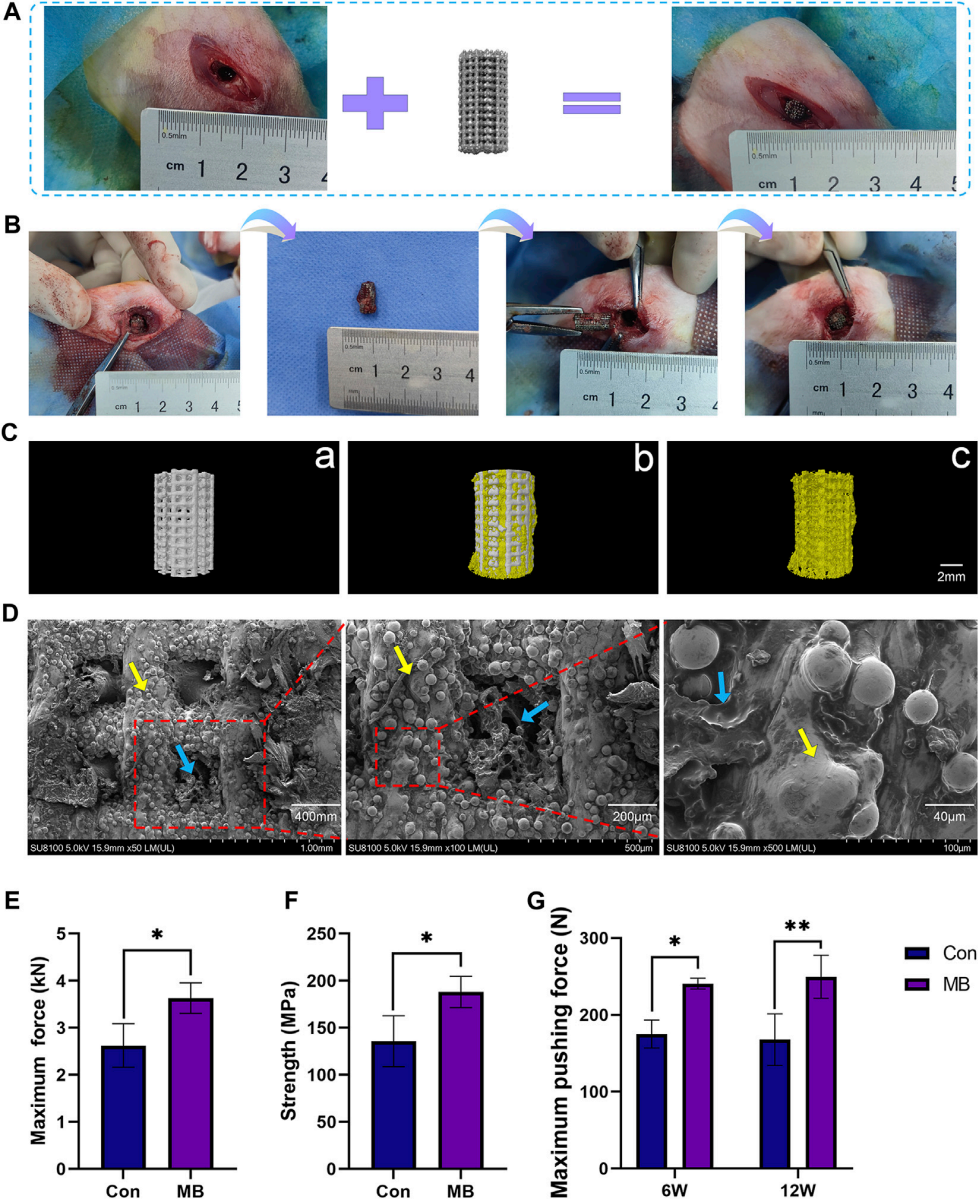
Two-Stage Surgical Procedure in Implantation. **(A)** Initial Implantation of Scaffolds. **(B)** Secondary implantation surgery procedure. **(C)** Micro-CT analysis: Representative 3D reconstruction image of a) the new scaffold (Con), b) the metal-bone scaffold (MB), c) the bone tissue inside the metal-bone scaffold. **(D)** SEM images of the bone tissue inside the metal-bone scaffolds (The area indicated by the yellow arrows is the Ti6Al4V scaffold region, and the area indicated by the blue arrows is the bone tissue region). **(E)** Compressive strength testing for the metal-bone scaffolds and new scaffolds (*n* = 3). **(F)** Maximum force in the biomechanical test of two types of scaffolds (*n* = 3). **(G)** Maximum pushing force of the Con and MB groups after implantation (*n* = 3). The data are expressed as the mean ± SD. *indicates significant differences between groups. *p < 0.05, **p < 0.01, and ***p < 0.001.

This animal experiment consisted of two surgeries. The first surgery involved the routine implantation of the orthopedic prosthesis, as illustrated in Figure 3A. After a period of 3 months, the scaffold had tightly integrated with the surrounding bone tissue. The second surgery was depicted in Figure 3B. First, the scaffold from the initial surgery was removed. In the MB group, the removed scaffold was re-implanted into the corresponding site on the contralateral limb. This group simulated the process of using the “metal-bone” scaffold for re-implantation, a less common clinical practice. In contrast, the control group also removed the scaffold but discarded it, then used an entirely new scaffold to be implanted in the same location on the contralateral limb. This control group simulated the more commonly used clinical strategy of re-implanting a new scaffold into the original site.

In Figure 3B, it can be observed that the removed prosthesis had integrated well with the surrounding bone tissue, and there was evident ingrowth of new bone tissue into the pores of the scaffold. Three-dimensional reconstruction based on Micro-CT results is illustrated in Figure 3C. A comparison with the plain Ti6Al4V scaffold (a) reveals that the “metal-bone” scaffold is filled with bone tissue in the pore. It shows a tight integration between the scaffold and bone tissue (b). Upon removing the Ti6Al4V scaffold section, abundant bone tissue (c) is observed within the scaffold pores. Quantitative analysis indicates that the bone tissue is approximately 41.68% ± 2.53% of the scaffold volume. This result validates the expected composition of the “metal-bone” scaffold, demonstrating the stable presence of internal tissue within the scaffold. Further observation of the bonding between bone tissue and the scaffold in “metal-bone” scaffolds was conducted using SEM, as depicted in Figure 3D. It is evident that the porous Ti6Al4V scaffold is filled with bone tissue, and there is a tight integration between the bone tissue and the scaffold surface. During the removal of the prosthesis in the second surgery, it was also noted that the scaffold had a strong integration effect with the bone tissue interface, resulting in significant resistance when pulling out the scaffold. The mechanical testing of the removed scaffold, as shown in Figures 3E, F, revealed that in MB group when the Ti6Al4V scaffold contained bone tissue, its strength significantly increased, and it could withstand greater forces. On one hand, this result indicates that the scaffold indeed contained a certain amount of bone tissue. On the other hand, it suggests that the bone tissue inside the “metal-bone” scaffold was tightly integrated with the scaffold, providing stronger mechanical support. This finding aligns with previous research, which also demonstrated that Ti6Al4V porous scaffolds exhibit excellent bone integration effects, especially with pore sizes in the range of 600–900 μm and a porosity of 70% (Taniguchi et al., 2016). The appropriate pore size facilitates the ingrowth of new bone tissue and nutrient supply, while a 70% porosity rate closely resembles the porosity rate of physiological bone trabeculae, making it a biomimetic physiological condition that achieves optimal bone integration effects (Pan et al., 2021).

To validate the osseointegration effectiveness between the scaffold and the surrounding bone tissue at the site of bone defects, mechanical push-out tests were conducted on the scaffold to measure the maximum force exerted when pushing the scaffold out of the bone tissue, as depicted in Figure 3G. It is evident that in the early stages of implantation, at 6 weeks, the MB group exhibited significantly greater bonding strength with the surrounding bone tissue in comparison to the control group, displaying a statistically significant difference (*p* < 0.5). By 12 weeks, the MB group also experienced a significantly higher maximum push-out force compared to the control group (*p* < 0.1). These findings suggest that the MB scaffold, which incorporates bone tissue, demonstrates enhanced osseointegration effects, characterized by higher bonding strength with the surrounding tissues when contrasted with the control group.

**FIGURE 4:**
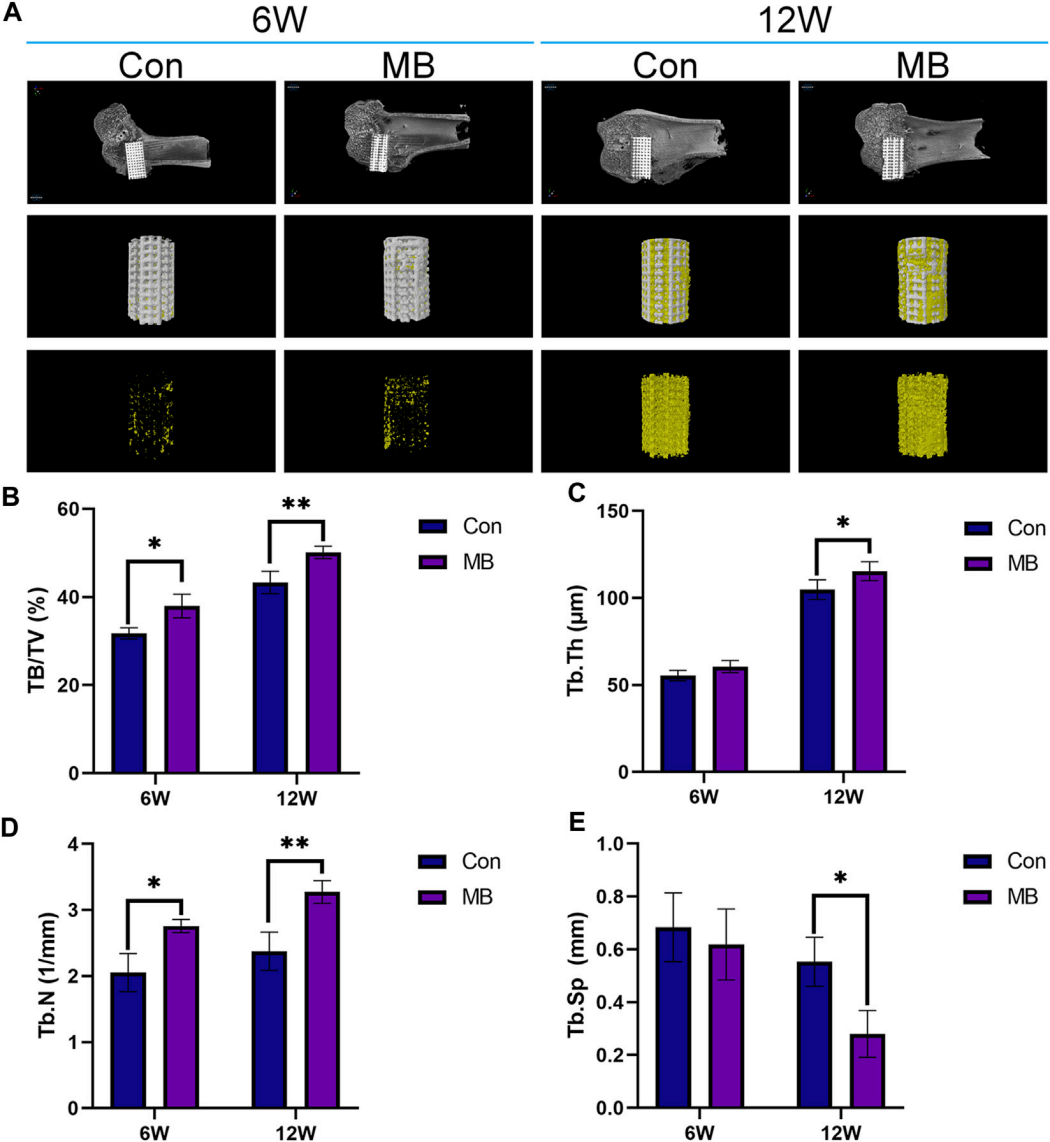
Micro-CT analysis of osseointegration around the prosthetic interfaces. **(A)** 3D reconstruction images around the prosthetic interfaces (The regenerated bone tissue is indicated in yellow and scaffolds are white). Quantitative analysis of **(B)** BV/TV, **(C)** Tb.Th, **(D)** Tb.N, and **(E)** Tb.Sp in two groups at 6 and 12 weeks after secondary implantation surgery (*n* = 3). The data are expressed as the mean ± SD. *indicates significant differences between groups. *p < 0.05, **p < 0.01, and ***p < 0.001.

In order to evaluate the formation of new bone in the control and MB groups, Micro-CT scans were conducted at 6 and 12 weeks, and the results are presented in Figure 4A. From the 3D reconstruction results, it is evident that at both 6 and 12 weeks, the scaffolds containing some bone tissue in the MB group, following the secondary surgery, had a greater amount of new bone tissue compared to the control group. Quantitative analysis, as shown in Figures 4B–E, indicates that the MB group had a higher proportion of regenerative bone tissue and a greater number of bone trabeculae compared to the control group.

From the Micro-CT results at 6 weeks, it is apparent that the MB group had a higher content of newly formed bone tissue than the control group. This suggests that the bone tissue contained within the “metal-bone” scaffold survived and integrated well with the surrounding tissues of the new bone defect after the revision surgery. After the secondary surgery, the Micro-CT results at 6 weeks indicate that the bone tissue in the “metal-bone” scaffold group is approximately 37.97% ± 2.67% (BV/TV), while the control group exhibits about 31.75% ± 1.29% bone tissue. The increased regenerated bone tissue in the “metal-bone” scaffold group is likely attributed to the survival of the original bone tissue within the scaffold. This surviving bone tissue contributes to accelerated bone defect repair by integrating with surrounding newly formed bone tissues, while the remaining internal bone tissues within the scaffold undergo absorption. Therefore, it can be inferred that the “metal-bone” scaffold containing some bone tissue accelerated the regeneration process following the initial prosthesis implantation, leading to faster bone repair. Moreover, the bone tissue within the “metal-bone” scaffold is the patient’s own tissue, eliminating the risk of immune rejection and allowing it to thrive.

**FIGURE 5:**
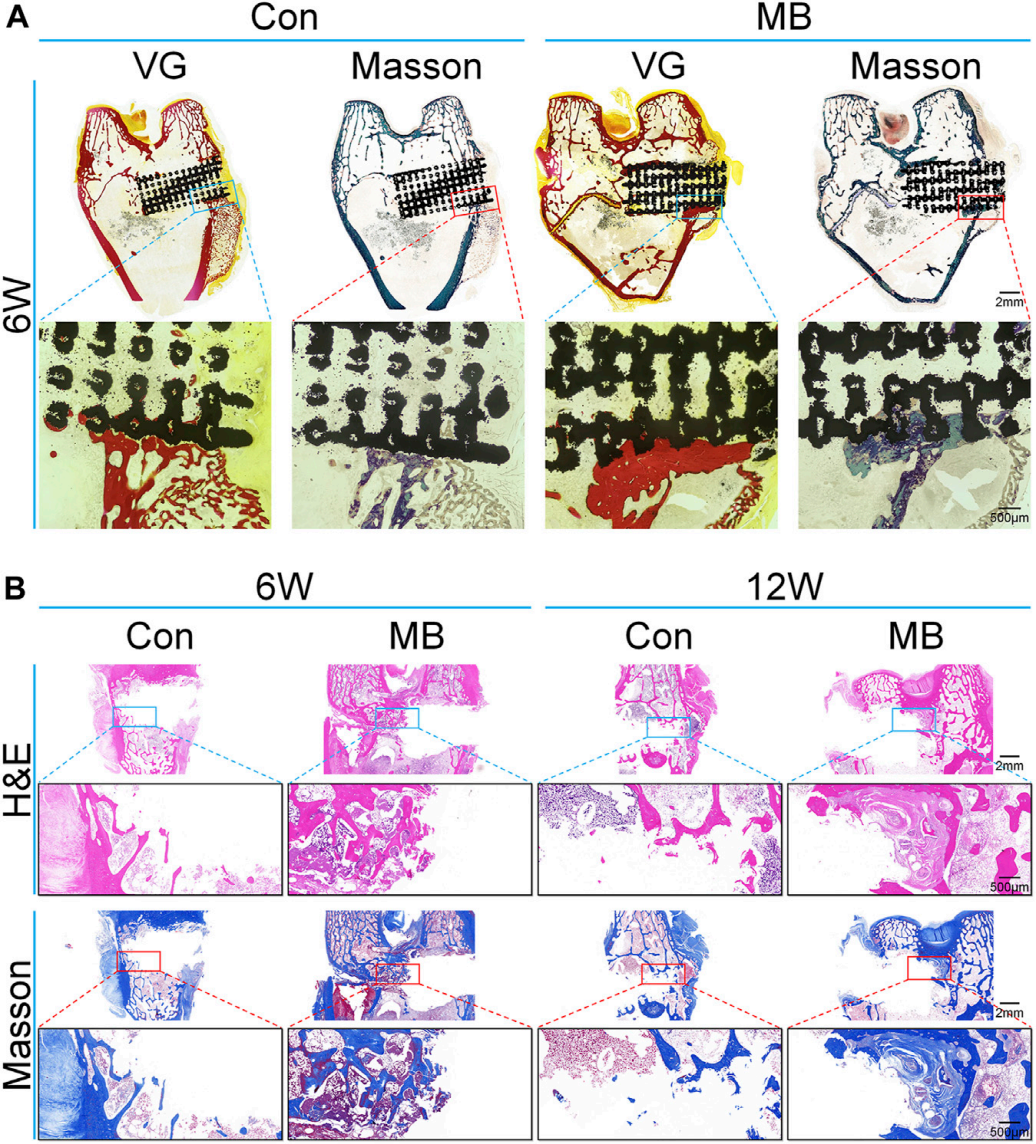
Histological analysis of bone regeneration around the scaffolds. **(A)** Van Gieson and Masson’s trichrome staining of the regenerated bone around scaffolds at 6 weeks (without removal of the scaffold). **(B)** H&E and Masson’s trichrome staining of the regenerated bone around scaffolds (after removal of the scaffold).

To get a clearer view of the bone regeneration within the scaffold, hard sectioning was performed on specimens obtained 6 weeks after the second surgery, as illustrated in Figure 5A. The black area represents the location of the scaffold. The red areas in blue VG and blue areas in Masson’s trichrome represents regenerated bones. Results from VG and Masson’s trichrome staining show that in the MB group, the depth of bone ingrowth around the scaffold was greater than that in the control group, and there was also a higher amount of newly formed bone tissue surrounding the scaffold, consistent with the Micro-CT results.

To further assess the bone regeneration at the site of bone defect, Ti6Al4V scaffolds were removed six and 12 weeks after the second surgery, followed by decalcification and sectioning. H&E staining as well as immunohistochemical staining for bone regeneration-related proteins were performed. The results for the bone tissue around the scaffold stained by H&E and Masson’s trichrome are shown in Figure 5B. From the H&E staining results, it can be observed that at 6 and 12 weeks, the bone trabeculae around the “metal-bone” scaffold containing bone tissue were more tightly integrated and there were more trabeculae compared to the control group. Masson’s staining reveals a higher content of newly formed bone tissue around the “metal-bone” scaffold, with active proliferation of bone trabeculae and the formation of more new bone trabeculae.

**FIGURE 6:**
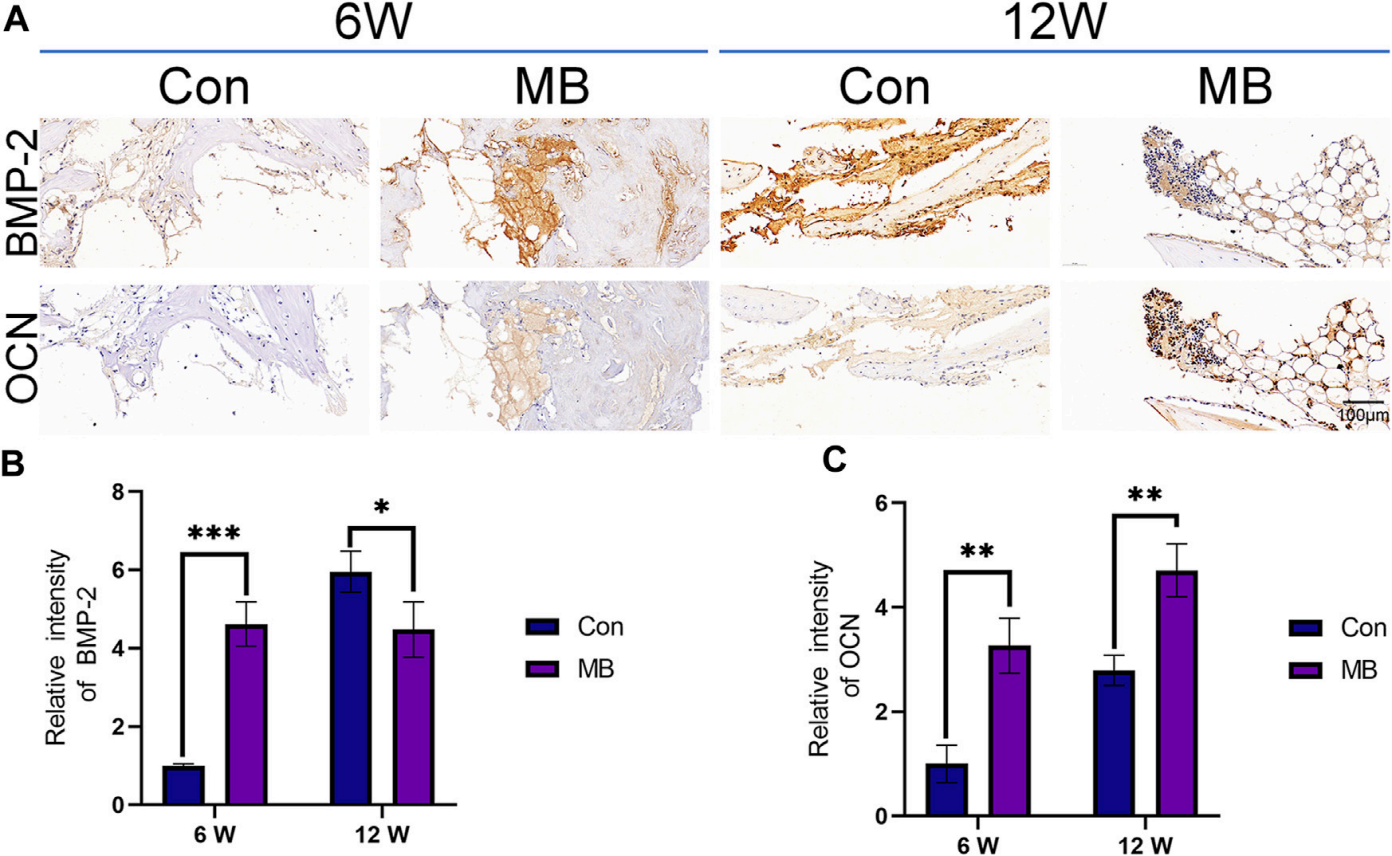
Immunohistochemical analysis of bone regeneration around the scaffolds. **(A)** Immunohistochemical staining of BMP-2 and OCN expression in each group. **(B)** Quantitative analysis of relative intensity of BMP-2 expression (*n* = 3). **(C)** Quantitative analysis of relative intensity of OCN expression (*n* = 3). The data are expressed as the mean ± SD. *indicates significant differences between groups. *p < 0.05, **p < 0.01, and ***p < 0.001.

The results of immunohistochemistry are presented in Figure 6A, with OCN protein being a hallmark protein for bone formation (Zhou et al., 2023), and BMP-2 being an important protein in the bone regeneration process (Wang et al., 2022). Immunohistochemical staining for these two proteins has been widely used in previous studies to assess bone regeneration strength (Santinoni et al., 2021; Fiorin et al., 2022).

From the immunohistochemical results of each group, it can be seen that at 6 weeks, the expression levels of both bone-forming marker proteins were significantly higher in the MB group than in the control group, as shown in Figures 6B, C. These differences were statistically significant. This suggests that in the MB group, the pre-existing bone tissue within the scaffold remained viable, leading to a faster bone formation process at the site of the bone defect. At this point, the bone regeneration effect at the bone defect site was superior to that in the control group.

By the 12th week, the expression level of OCN protein in the MB group remained higher than that in the control group, but the expression of BMP-2 was lower than that in the control group. Considering that BMP-2 is an osteogenic growth factor that promotes bone formation (Zhang et al., 2019; Bi et al., 2023), this result suggests that the strong bone-promoting effect in the control group was achieved at 12 weeks, later than in the MB group.

The analysis of the animal experiment results leads to the conclusion that the MB scaffold containing some bone tissue is superior to a new scaffold. The reason for this could be the presence of host bone within the original scaffold. When re-implanted into the bone defect site, the tissue inside the scaffold can survive and integrate with the surrounding tissues, thereby accelerating the bone regeneration process. Compared to a new scaffold, the bone tissue around the MB scaffold can initiate the bone formation process more quickly, facilitating the migration of BMSCs and osteogenic cells to the scaffold site, thereby reducing the time required for bone repair and achieving better results.

In this study, we have combined the widely used Ti6Al4V scaffold in bone tissue engineering with clinical bone transplantation techniques to fabricate a “metal-bone” scaffold, which exhibits superior osteoinductive properties compared to pure Ti6Al4V scaffolds, thus accelerating bone healing. Ti6Al4V scaffolds are widely adopted in bone tissue engineering due to their excellent mechanical strength, biocompatibility, and osseointegration (Liao et al., 2021). However, their high mechanical strength often leads to stress shielding phenomena. To overcome this limitation, porous structures are commonly produced using 3D printing technology. On one hand, these porous structures mitigate stress shielding effects, while micro-porosities promote bone tissue ingrowth, enhance bone formation, and aid in scaffold fixation. Nevertheless, due to the biologically inert nature of Ti6Al4V, efficient bone regeneration often necessitates the incorporation of bioactive substances into porous scaffolds, especially in the case of larger bone defects (Koju et al., 2022).

Considering the clinical challenges associated with treating substantial bone defects, autografting is a well-established approach. However, in cases of extensive bone loss, autograft availability may be limited, and the excessive use of autografts can lead to iatrogenic damage, while allografts may trigger immune rejection responses (Baldwin et al., 2019). In this study, we have innovatively combined 3D-printed porous Ti6Al4V scaffolds with autografting techniques. Our findings confirm that the addition of a small amount of autograft within the porous scaffold can efficiently promote bone regeneration. Furthermore, our animal experiments demonstrate that the “metal-bone” scaffold outperforms plain porous Ti6Al4V scaffolds by expediting early-stage bone regeneration, thus reducing the time required for peri-implant endosseous healing. This research provides valuable clinical insights, substantiating the effectiveness of incorporating a small amount of viable autograft around Ti6Al4V scaffolds to accelerate bone healing and facilitate osteogenesis.

Compared to clinical implants, our study innovatively employs a 3D-printed porous Ti6Al4V scaffold, with its design parameters informed by prior research findings to optimize osteogenesis. Additionally, we integrate clinical bone grafting techniques with porous titanium alloy scaffolds in this study, creating a novel “Metal-Bone” scaffold through implantation. This scaffold contains bioactive bone tissue internally, and observations indicate a tight integration between the scaffold and its internal bone tissue. Following implantation into bone defects, it significantly enhances osteointegration, accelerates bone healing compared to titanium alloy scaffolds without bone tissue, promoting the integration of the implant with the surrounding bone tissues. In terms of clinical applications, considering the substantial extraction of bone tissue during orthopedic implant surgeries (Wazzan et al., 2023), incorporating a small amount of autologous bone tissue into implants is conceivable. This strategy has the potential to accelerate bone regeneration and strengthen the bone integration around the implanted prosthetic. The primary challenges lie in whether the bone tissue obtained during replacement surgery can maintain its vitality after fragmentation. Additionally, there is a requirement for the implant to be porous to accommodate sufficient bone tissue. In clinical translation, numerous challenges will be encountered, requiring further in-depth investigation for the potential clinical application of the “Metal-Bone” scaffold.

Furthermore, the “metal-bone” scaffold employed in this study shares similarities with the old scaffolds removed during orthopedic revision surgeries. Consequently, this research also furnishes empirical evidence regarding the choice between using old prostheses containing some bone tissue or opting for entirely new prostheses in orthopedic revision procedures.

Joint replacement surgery is one of the top five most common surgeries annually and one of the top five fastest-growing procedures (Schwartz et al., 2020; Pigeolet et al., 2021). The increasing number of patients receiving prosthetic joint replacement surgery is driven by factors such as osteoporosis, trauma, and tumors (Hunter and Bierma-Zeinstra, 2019). As the number of joint replacement surgeries rises, so do postoperative complications, including prosthesis sinking, loosening, and displacement (Howard et al., 2023). Currently, over 30% of joint replacement patients undergo one or more joint replacement surgeries in 20 years (Lee et al., 2023). In clinical practice, when addressing postoperative complications following joint replacement, it is common to replace the old prosthesis with a new one during revision surgery (Gong et al., 2023). However, intraoperatively, it is often observed that the original prosthesis remains intact, with complications primarily arising due to inadequate bonding between the prosthesis and surrounding bone tissue. Using new prostheses in revision surgery not only results in the wastage of medical materials but also imposes an economic burden on patients and their families (Cimatti et al., 2022). Currently, there is no definitive research comparing the efficacy of retaining the original prosthesis versus using a new one in revision surgery. This study addresses this clinical issue through experimental animal research by comparing the outcomes of these two prosthesis approaches. The results confirm that, in orthopedic revision surgeries, prostheses containing some bone tissue, when still viable, exhibit osteoinductive properties similar to new prostheses. Consequently, this study offers a novel perspective to some extent for revision surgeries, carrying certain clinical implications.

## 4 Conclusion

In summary, this study combines 3D-printed porous Ti6Al4V prostheses commonly used in bone tissue engineering with clinical bone transplantation techniques to create a novel “metal-bone” scaffold for promoting bone regeneration in cases of bone defects. Firstly, the scaffold’s consistency with expectations was validated through SEM, EDS, and similar methods. Subsequently, cell experiments confirmed the scaffold’s biocompatibility. Finally, through animal experiments involving a two-stage surgical approach, wherein a “metal-bone” scaffold with bone tissue was first prepared over 3 months and then implanted into the contralateral limb bone defect, it was compared against a new scaffold lacking bone tissue. The results substantiated the survival of bone tissue within the “metal-bone” scaffold post-transplantation, achieving more effective bone regeneration and expediting the healing process. This research opens up significant prospects for the clinical application of orthopedic prostheses preparation and revision surgeries.

## Data Availability

The original contributions presented in the study are included in the article/Supplementary material, further inquiries can be directed to the corresponding authors.

## References

[B1] Abbasi N. Hamlet S. Love R. M. Nguyen N.-T. (2020). Porous scaffolds for bone regeneration. J. Sci. Adv. Mater. Devices 5 (1), 1–9. 10.1016/j.jsamd.2020.01.007

[B2] Altunbek M. Afghah S. F. Fallah A. Acar A. A. Koc B. (2023). Design and 3D printing of personalized hybrid and gradient structures for critical size bone defects. ACS Appl. Bio Mater 6 (5), 1873–1885. 10.1021/acsabm.3c00107 PMC10189796 37071829

[B3] Bai H. Zhao Y. Wang C. Wang Z. Wang J. Liu H. (2020). Enhanced osseointegration of three-dimensional supramolecular bioactive interface through osteoporotic microenvironment regulation. Theranostics 10 (11), 4779–4794. 10.7150/thno.43736 32308749 PMC7163459

[B4] Baldwin P. Li D. J. Auston D. A. Mir H. S. Yoon R. S. Koval K. J. (2019). Autograft, allograft, and bone graft substitutes: clinical evidence and indications for use in the setting of orthopaedic trauma surgery. J. Orthop. Trauma 33 (4), 203–213. 10.1097/BOT.0000000000001420 30633080

[B5] Bi Z. Shi X. Liao S. Li X. Sun C. Liu J. (2023). Strategies of immobilizing BMP-2 with 3D-printed scaffolds to improve osteogenesis. Regen. Med. 18 (5), 425–441. 10.2217/rme-2022-0222 37125508

[B6] Calore A. R. Srinivas V. Groenendijk L. Serafim A. Stancu I. C. Wilbers A. (2023). Manufacturing of scaffolds with interconnected internal open porosity and surface roughness. Acta Biomater. 156, 158–176. 10.1016/j.actbio.2022.07.017 35868592

[B7] Chen Z. Yan X. Yin S. Liu L. Liu X. Zhao G. (2020). Influence of the pore size and porosity of selective laser melted Ti6Al4V ELI porous scaffold on cell proliferation, osteogenesis and bone ingrowth. Mater Sci. Eng. C Mater Biol. Appl. 106, 110289. 10.1016/j.msec.2019.110289 31753386

[B8] Cimatti P. Andreoli I. Busacca M. Govoni M. Vivarelli L. Del Piccolo N. (2022). An observational prospective clinical study for the evaluation of a collagen-hydroxyapatite composite scaffold in hip revision surgery. J. Clin. Med. 11 (21), 6372. 10.3390/jcm11216372 36362601 PMC9654158

[B9] Cui Y. Wang Z. Li Z. Ji X. Yuan B. Sun Y. (2021). Functionalized anti-osteoporosis drug delivery system enhances osseointegration of an inorganic–organic bioactive interface in osteoporotic microenvironment. Mater. Des. 206, 109753. 10.1016/j.matdes.2021.109753

[B10] Fiorin L. G. Matheus H. R. Ervolino E. Canciani E. Pellegrini G. Dellavia C. (2022). Tamoxifen improves homeostasis in the peri-implant bone remodeling of osseointegrated titanium implants. J. Periodontal Res. 57 (4), 880–890. 10.1111/jre.13026 35856857

[B11] Gong L. Chen X. Shao H. Gu J. Dong R. Ding Y. (2023). Clinicopathological analysis of the periprosthetic tissue of revision total hip and knee arthroplasty. Chin. Med. J. Engl. 136 (15), 1870–1872. 10.1097/CM9.0000000000002219 37358527 PMC10406048

[B12] Gu Y. Sun Y. Shujaat S. Braem A. Politis C. Jacobs R. (2022). 3D-printed porous Ti6Al4V scaffolds for long bone repair in animal models: a systematic review. J. Orthop. Surg. Res. 17 (1), 68. 10.1186/s13018-022-02960-6 35109907 PMC8812248

[B13] Henkel J. Medeiros Savi F. Berner A. Fountain S. Saifzadeh S. Steck R. (2021). Scaffold-guided bone regeneration in large volume tibial segmental defects. Bone 153, 116163. 10.1016/j.bone.2021.116163 34461285

[B14] Howard L. C. Day C. W. Masri B. A. Garbuz D. S. (2023). Comparison of clinical and functional outcomes in one versus two component revision for total knee arthroplasty. J. Arthroplasty 38 (6S), S275–S280. 10.1016/j.arth.2023.01.047 36739924

[B15] Hunter D. J. Bierma-Zeinstra S. (2019). Osteoarthritis. Lancet 393 (10182), 1745–1759. 10.1016/S0140-6736(19)30417-9 31034380

[B16] Jockusch B. M. Rothkegel M. Schwarz G. (2004). Linking the synapse to the cytoskeleton: a breath-taking role for microfilaments. Neuroreport 15 (10), 1535–1538. 10.1097/01.wnr.0000131673.92694.58 15232278

[B17] Koju N. Niraula S. Fotovvati B. (2022). Additively manufactured porous Ti6Al4V for bone implants: a review. Metals 12 (4), 687. 10.3390/met12040687

[B18] Lee J. J. Oladeji K. Sweeney B. F. Chakoma T. L. Arora P. Finlay A. K. (2023). Single, recurrent, synchronous, and metachronous periprosthetic joint infections in patients with multiple hip and knee arthroplasties. J. Arthroplasty 38 (9), 1846–1853. 10.1016/j.arth.2023.03.014 36924855 PMC11465106

[B19] Li S. Lei H. Liu H. Song P. Fan S. Wu L. (2023). Pulsed electrodeposition of MXenes/HAp multiple biological functional coatings on 3D printed porous Ti-6Al-4V bone tissue engineering scaffold. Surf. Coatings Technol. 464, 129532. 10.1016/j.surfcoat.2023.129532

[B20] Liao B. Xia R. F. Li W. Lu D. Jin Z. M. (2021). 3D-Printed Ti6Al4V scaffolds with graded triply periodic minimal surface structure for bone tissue engineering. J. Mater. Eng. Perform. 30 (7), 4993–5004. 10.1007/s11665-021-05580-z

[B21] Liu B. Hou G. Yang Z. Li X. Zheng Y. Wen P. (2022). Repair of critical diaphyseal defects of lower limbs by 3D printed porous Ti6Al4V scaffolds without additional bone grafting: a prospective clinical study. J. Mater Sci. Mater Med. 33 (9), 64. 10.1007/s10856-022-06685-0 36104513 PMC9474430

[B22] Montero J. Fernandez-Ruiz A. Pardal-Pelaez B. Jimenez-Guerra A. Velasco-Ortega E. Nicolas-Silvente A. I. (2020). Effect of rough surface platforms on the mucosal attachment and the marginal bone loss of implants: a dog study. Mater. (Basel) 13 (3), 802. 10.3390/ma13030802 PMC7040816 32050603

[B23] Muranova L. K. Shatov V. M. Gusev N. B. (2022). Role of small heat shock proteins in the remodeling of actin microfilaments. Biochem. (Mosc) 87 (8), 800–811. 10.1134/S0006297922080119 36171660

[B24] Naghavi S. A. Tamaddon M. Garcia-Souto P. Moazen M. Taylor S. Hua J. (2023a). A novel hybrid design and modelling of a customised graded Ti-6Al-4V porous hip implant to reduce stress-shielding: an experimental and numerical analysis. Front. Bioeng. Biotechnol. 11, 1092361. 10.3389/fbioe.2023.1092361 36777247 PMC9910359

[B25] Naghavi S. A. Tamaddon M. Garcia-Souto P. Moazen M. Taylor S. Hua J. (2023b). A novel hybrid design and modelling of a customised graded Ti-6Al-4V porous hip implant to reduce stress-shielding: an experimental and numerical analysis. Front. Bioeng. Biotechnol. 11, 1092361. 10.3389/fbioe.2023.1092361 36777247 PMC9910359

[B26] Nayak V. V. Slavin B. Bergamo E. T. P. Boczar D. Slavin B. R. Runyan C. M. (2023). Bone tissue engineering (BTE) of the craniofacial skeleton, Part I: evolution and optimization of 3D-printed scaffolds for repair of defects. J. Craniofac Surg. 34 (7), 2016–2025. 10.1097/SCS.0000000000009593 37639650 PMC10592373

[B27] Pan C. T. Hsu W. H. Cheng Y. S. Wen Z. H. Chen W. F. (2021). A new design of porosity gradient Ti-6Al-4V encapsulated hydroxyapatite dual materials composite scaffold for bone defects. Micromachines (Basel) 12 (11), 1294. 10.3390/mi12111294 34832706 PMC8624878

[B28] Pigeolet M. Jayaram A. Park K. B. Meara J. G. (2021). Osteoarthritis in 2020 and beyond. Lancet 397 (10279), 1059–1060. 10.1016/S0140-6736(21)00208-7 33743863

[B29] Santinoni C. S. Neves A. P. C. Almeida B. F. M. Kajimoto N. C. Pola N. M. Caliente E. A. (2021). Bone marrow coagulated and low-level laser therapy accelerate bone healing by enhancing angiogenesis, cell proliferation, osteoblast differentiation, and mineralization. J. Biomed. Mater Res. A 109 (6), 849–858. 10.1002/jbm.a.37076 32815657

[B30] Schwartz A. M. Farley K. X. Guild G. N. Bradbury T. L. Jr. (2020). Projections and epidemiology of revision hip and knee arthroplasty in the United States to 2030. J. Arthroplasty 35 (6S), S79–S85. 10.1016/j.arth.2020.02.030 32151524 PMC7239745

[B31] Sokoot E. A. Arkan E. Khazaei M. Moradipour P. (2023). A novel 3D-electrospun nanofibers-scaffold grafted with Royal Jelly: improve hydrophilicity of the nanofibers-scaffold and proliferation of HUVEC cell line. Cell. Tissue Bank. 24 (2), 329–340. 10.1007/s10561-022-10035-3 36284047

[B32] Subasi O. Karaismailoglu B. Ashkani-Esfahani S. Lazoglu I. (2023). Investigation of lattice infill parameters for additively manufactured bone fracture plates to reduce stress shielding. Comput. Biol. Med. 161, 107062. 10.1016/j.compbiomed.2023.107062 37235944

[B33] Taniguchi N. Fujibayashi S. Takemoto M. Sasaki K. Otsuki B. Nakamura T. (2016). Effect of pore size on bone ingrowth into porous titanium implants fabricated by additive manufacturing: an *in vivo* experiment. Mater Sci. Eng. C Mater Biol. Appl. 59, 690–701. 10.1016/j.msec.2015.10.069 26652423

[B34] Wang C. Huang W. Zhou Y. He L. He Z. Chen Z. (2020). 3D printing of bone tissue engineering scaffolds. Bioact. Mater 5 (1), 82–91. 10.1016/j.bioactmat.2020.01.004 31956737 PMC6962643

[B35] Wang C. Xu D. Lin L. Li S. Hou W. He Y. (2021a). Large-pore-size Ti6Al4V scaffolds with different pore structures for vascularized bone regeneration. Mater Sci. Eng. C Mater Biol. Appl. 131, 112499. 10.1016/j.msec.2021.112499 34857285

[B36] Wang J. Wei Y. Zhou Z. Yang J. Jia Y. Wu H. (2022). Deer antler extract promotes tibia fracture healing in mice by activating BMP-2/SMAD4 signaling pathway. J. Orthop. Surg. Res. 17 (1), 468. 10.1186/s13018-022-03364-2 36307889 PMC9617435

[B37] Wang X. Li Z. Wang Z. Liu H. Cui Y. Liu Y. (2021b). Incorporation of bone morphogenetic protein-2 and osteoprotegerin in 3D-printed Ti6Al4V scaffolds enhances osseointegration under osteoporotic conditions. Front. Bioeng. Biotechnol. 9, 754205. 10.3389/fbioe.2021.754205 34805113 PMC8600075

[B38] Wang Z. Wang C. Li C. Qin Y. Zhong L. Chen B. (2017). Analysis of factors influencing bone ingrowth into three-dimensional printed porous metal scaffolds: a review. J. Alloys Compd. 717, 271–285. 10.1016/j.jallcom.2017.05.079

[B39] Wazzan A. L. J. Almusallam M. H. Almosa M. S. Bin Dukhi M. M. Bin Akrish A. M. Alaraidh S. A. (2023). Etiologies of orthopedic implant removal among patients who underwent orthopedic fixation surgeries in king abdulaziz medical city. Cureus 15 (8), e43809. 10.7759/cureus.43809 37731419 PMC10508870

[B40] Zeng S. Liu G. Li C. Ye J. Li D. (2022). Porous structure design and mechanical properties analysis of femoral stem based on selective laser melting. Chin. J. Lasers-Zhongguo Jiguang 49 (2), 0202016. 10.3788/cjl202249.0202016

[B41] Zhang X. Lou Q. Wang L. Min S. Zhao M. Quan C. (2019). Immobilization of BMP-2-derived peptides on 3D-printed porous scaffolds for enhanced osteogenesis. Biomed. Mater 15 (1), 015002. 10.1088/1748-605X/ab4c78 31597124

[B42] Zhang Y. Sun N. Zhu M. Qiu Q. Zhao P. Zheng C. (2022). The contribution of pore size and porosity of 3D printed porous titanium scaffolds to osteogenesis. Biomater. Adv. 133, 112651. 10.1016/j.msec.2022.112651 35034817

[B43] Zhou Y. Zhu P. Shen S. Wang Y. Li B. Guo B. (2023). Overexpression of fibroblast growth factor receptor 2 in bone marrow mesenchymal stem cells enhances osteogenesis and promotes critical cranial bone defect regeneration. Front. Cell. Dev. Biol. 11, 1208239. 10.3389/fcell.2023.1208239 37266455 PMC10229770

